# The Association of Season and Temperature with Adverse Pregnancy Outcome in Two German States, a Time-Series Analysis

**DOI:** 10.1371/journal.pone.0040228

**Published:** 2012-07-06

**Authors:** Jennyfer Wolf, Ben Armstrong

**Affiliations:** Department of Social and Environmental Health, London School of Hygiene and Tropical Medicine, London, United Kingdom; University of Montreal, Canada

## Abstract

**Background:**

A seasonality of low birth weight (LBW) and preterm birth (PTB) has been described for most regions and there is evidence that this pattern is caused by ambient outdoor temperature. However, the association as such, the direction of effect and the critical time of exposure remain controversial.

**Methods:**

Logistic, time-series regression was performed on nearly 300,000 births from two German states to study the association between season and daily mean temperature and changes in daily proportions of term LBW (tLBW) or PTB. Analyses were adjusted for time-varying factors. Temperature exposures were examined during different periods of pregnancy.

**Results:**

Weak evidence for an association between season of conception, season of birth or ambient outdoor temperature and tLBW or PTB was found. Results of analyses of temperature were not consistent between the two states. Different sources of bias which would have artificially led to stronger findings were detected and are described.

**Conclusions:**

No clear evidence for an association between season of conception, season of birth or temperature and tLBW or PTB was found. In the study of pregnancy outcome different sources of bias can be identified which can potentially explain heterogeneous findings of the past.

## Introduction

Low birth weight (LBW) and preterm birth (PTB) are major contributors to short- and long-term morbidity [1], [2] and mortality [3]–[5]. Yet, their causes are largely unknown [6]. A seasonal pattern has been described for both LBW [7]–[13] and PTB [14]–[17] with major interregional differences [6], [18]. As temperature varies with season, it is a potential determinant for this pattern.

High temperature was associated with both conditions: A birth cohort study in Scotland described reduced birth weight when exposure to high temperature occurred early in pregnancy [7]. A between-population analysis detected a significant inverse relation between heat and birth weight in 108 out of 140 populations [19]. This finding was confirmed by a U.S. study on 37 million births [20]. In Greece high temperature around the day of birth was correlated with both reduced birth weight and shorter gestational age [21]. In a case-crossover analysis in California a significant increase in PTB with higher temperature in the week before birth was detected [22]. In New York and Barcelona an association between heat and humidity and preterm labour or reduced gestational age was seen [23], [24].

Also cold has been associated with reduced weight at birth when exposure occurred during mid-pregnancy [25], [26] or in the third trimester [7].

Evidence from animal studies supports the hypothesis of an association between temperature and birth weight [27]–[30] and suggests a detrimental effect of heat on the developing embryo [31], [32]. Also length of gestation showed seasonal variability in different animals [33]–[35].

How temperature might act on the foetus remains relatively unclear. Reduced body size was seen as adaption to heat through a high surface-to-volume ratio [36]–[38]. Cold exposure was thought more plausibly to hinder normal growth [26].

The overall aim of this study was to examine a potential seasonal rhythm of adverse pregnancy outcome in two German populations and to add to the quantification of current and possible future health impacts of temperature. The main research hypothesis was that tLBW and PTB show seasonal patterns which can be explained by differences in temperature.

## Materials and Methods

### Ethics Statement

Approval of this study was granted by the Research Ethics Committee of the London School of Hygiene and Tropical Medicine. As all potential identifiers were removed from the data, no written informed consent from the participants of the study was necessary.

Two datasets with daily birth records were obtained from the Departments of Quality Management of the State Chambers of Physicians of Brandenburg (years 2002 to 2010) and Saxony (2005 to 2009). The data was originally collected for the German “Perinatalerhebung” which contains all reported hospital births from these two states.

Only singletons with a gestational age between 20 and 44 weeks and a birth weight greater than 200 g were included. Gestational age was counted from first day of last menstrual period. Gestational weeks are stated in completed weeks. Date of conception was defined as 14 days after date of last menstrual period.

LBW, defined as birth weight below 2500 g, can result both from PTB and intrauterine growth restriction [39], [40]. To avoid overlapping with PTB, LBW, as commonly done before [25], [26], [41], was restricted to term births (≥37 weeks of gestation).

PTB was defined as birth before 37 gestational weeks. As done before [41], early temperature exposures were examined on all PTBs whereas for the late exposure only PTBs of at least 29 weeks were considered. The underlying assumption was that an exposure which occurs late in pregnancy does not lead to induction of very early PTB.

Time-series data on daily mean temperature for eight weather monitoring stations in Brandenburg (five stations) and Saxony (four stations) was obtained from the German Meteorological Service. The temperature data from these stations was highly correlated by state (Pearson’s correlation coefficient between 0.94 and 1.0, examined was overall temperature correlation and correlation separately by season) and mean temperature was very similar (mean temperature ranged in Brandenburg between 9.1 and 9.6°C and in Saxony between 9.0 and 9.7°C). Therefore we chose one weather monitoring station for each state (Potsdam and Dresden).

Logistic time-series regression was used to examine both seasonality and the relationship between daily mean temperature and daily proportions of tLBW and PTB.

To examine seasonality, indicator terms for seasons (defined a-priori as December to February: winter, March to May: spring, June to August: summer, September to November: autumn) and for individual months adjusted for calendar year were used. Sensitivity analysis was performed with Fourier (trigonometric) terms with one sine-cosine pattern with annual periodicity [42].

To examine temperature and to allow for both acute and delayed effects, a constrained distributed lag model was used, whereby temperature was averaged over different time periods of pregnancy [43]. These periods were defined a-priori relating to biological plausibility and in accordance with other researchers [7], [41]. For the study of tLBW, mean temperature during the three trimesters of pregnancy was investigated. To examine PTB, mean temperature during the first month, the first trimester and the last week before birth was studied.

Temperature effects on health might follow a linear or, more likely, a non-linear, U- or V-shaped relationship with increased effects at both low and high values [43], [44]. We analysed temperature both as a linear and a categorical variable. Analyses of temperature were adjusted for both seasonal changes and long-term trend with indicator terms for calendar months and year or, alternatively, with smooth functions of time (B-splines) with two to four knots per year. The Akaike’s information criterion was used to compare the model fit [43]. The full model by date of birth was further adjusted for day of the week which is a known predictor of adverse pregnancy outcome [45].

When pregnancy data is collected by date of birth but analysed by date of conception, longer pregnancies will dominate at the start and shorter pregnancies at the end of data collection. This phenomenon has been described before [46] and arises when short pregnancies that were conceived on the earliest potential conception dates have already terminated before the start of the inclusion period and will therefore not appear in the analysis. The same principle applies to the end of the dataset when long pregnancies from the latest possible conception dates will only end after the last birth is included in the study. Thereby, the earliest conception dates will appear at greater risk for shorter pregnancies whereas the latest will seem protective. This might lead to false associations with a time-varying factor like temperature. To prevent this truncation bias, we restricted the inclusion of pregnancies in analyses by date of conception, i.e. we excluded pregnancies conceived earlier than 37 weeks (tLBW) or 20 weeks (PTB) before the study start date or later than 44 weeks before the study end date [46]. As a result all gestational ages are included throughout the study period.

**Table 1:** Characteristics of births in Brandenburg (n = 128,604) and Saxony (n = 162,913).

Brandenburg	N (%)		N (%)
Preterm birth	8,717 (6.8)	Year of birth	
Low birth weight	6,242 (4.9)	2002	13,482 (10.5)
Female babies	62,985 (49.0)	2003	13,805 (10.7)
Season of birth		2004	14,688 (11.4)
Dec–Feb	29,787 (23.2)	2005	14,345 (11.2)
Mar–May	31,361 (24.4)	2006	14,371 (11.2)
Jun–Aug	35,282 (27.4)	2007	14,718 (11.4)
Sep–Nov	32,174 (25.0)	2008	14,388 (11.2)
		2009	14,331 (11.1)
		2010	14,476 (11.3)
**Saxony**	**N (%)**		**N (%)**
Preterm birth	10,277 (6.3)	Year of birth	
Low birth weight	8,034 (4.9)	2005	31,576 (19.4)
Female babies	79,168 (48.6)	2006	31,708 (19.5)
Season of birth		2007	32,999 (20.3)
Dec–Feb	38,181 (23.4)	2008	33,746 (20.7)
Mar–May	39,309 (24.1)	2009	32,884 (20.2)
Jun–Aug	44,264 (27.2)		
Sep–Nov	41,159 (25.3)		

**Table 2:** Seasonality of tLBW and PTB by date of conception (doc) and birth (dob).

			Brandenburg			Saxony		
		Adjusted for	OR	(95% CI)	p-value	OR	(95% CI)	p-value
tLBW by doc		year			Wald-test			Wald-test
Seasons					0.02			0.8
	Winter		1.06	(0.93,1.21)		0.98	(0.88,1.10)	
	Spring		1.19	(1.05,1.35)		1.04	(0.93,1.16)	
	Summer		1			1		
	Autumn		1.00	(0.88,1.13)		0.98	(0.88,1.09)	
Fourier					0.02			0.2
Months					0.1			0.5
**tLBW by dob**		**year, day of week**						
Seasons					0.1			0.7
	Winter		1.15	(1.02,1.30)		1.01	(0.90,1.12)	
	Spring		1.07	(0.94,1.21)		0.96	(0.86,1.07)	
	Summer		1			1		
	Autumn		1.13	(1.00,1.28)		0.96	(0.86,1.06)	
Fourier					0.02			0.6
Months					0.07			0.7
**PTB by doc**		**year**						
Seasons					0.06			0.3
	Winter		1.06	(0.99,1,13)		1.00	(0.94,1.07)	
	Spring		1.08	(1.01,1.15)		1.04	(0.98,1.11)	
	Summer		1			1		
	Autumn		1.01	(0.95,1.08)		0.98	(0.92,1.05)	
Fourier					0.05			0.01
Months					0.09			0.09
**PTB by dob**		**year, day of week, gestational age, pregnancies-at-risk approach**						
Seasons					0.4			0.07
	Winter		0.98	(0.92,1.04)		1.07	(1.01,1.13)	
	Spring		0.97	(0.92,1.03)		1.01	(0.96,1.07)	
	Summer		1			1		
	Autumn		1.03	(0.96,1.09)		1.06	(1.00,1.12)	
Fourier					0.1			0.05
Months					0.3			0.1

Baseline ORs in this table are placed at season with lowest risk.

For analyses by date of conception, the numerator of the estimated proportions included all tLBW- or preterm births resulting from conceptions on each day and the denominator included all conceptions on that day. For analyses of tLBW by date of birth, the numerator included all tLBW births on each day and the denominator all term births on that day. For analyses of PTB by date of birth a pregnancies-at-risk approach [15] was applied. In this approach the denominator includes all pregnancies at risk of being born preterm on that day; that is, pregnancies from the 29^th^ to 36^th^ week of gestation. This takes into account that after the 36^th^ week a pregnancy belongs no longer to this risk-set. This is necessary because the number of pregnancies at-risk and also their distribution of gestational ages show a seasonal pattern because the number of births and conceptions varies regularly throughout the year [14], [15], [47]. Finally, we control for pregnancies between week 29 and 36 having very different probabilities to end preterm, with a strong increase in higher gestational ages. A higher proportion of PTB at a particular time can without such control arise solely because pregnancies in the risk-set have a higher mean gestational age [15]. Therefore, pregnancies at-risk were stratified by gestational age in weeks [15], [41] and gestational age was additionally included in the model as an explanatory indicator variable.

Information on maternal age was only available for Saxony. As it was described before that maternal characteristics that show seasonal patterns can confound an association between season and adverse pregnancy outcome and as maternal age showed such a pattern in our dataset, we re-analysed the Saxony data with the inclusion of maternal age (three groups <20 years, 20–34 years, ≥35 years [48], [49]) as an explanatory variable.

Deviance residuals of the full model were plotted against date and autocorrelation was examined with the Partial Autocorrelation Function (PACF). Analyses were performed using Stata 11 software (StataCorp. 2009. Stata Statistical Software: Release 11. College Station, TX: StataCorp LP).

**Table 3:** Association between temperature and tLBW or PTB in Brandenburg.

tLBW		1^st^ trimester			2^nd^ trimester			3^rd^ trimester		
		OR	(95% CI)	p	OR	(95% CI)	p	OR	(95% CI)	p
adjusted:		month, year			month, year			month, year		
Quintile										
	1	1		0.7	1		0.05	1		0.5
	2	1.02	(0.86, 1.21)		0.85	(0.70,1.02)		0.86	(0.71, 1.05)	
	3	1.18	(0.90, 1.53)		0.68	(0.51,0.91)		0.95	(0.70, 1.28)	
	4	1.14	(0.81, 1.63)		0.70	(0.48,1.00)		1.04	(0.70, 1.55)	
	5	1.10	(0.73, 1.66)		0.81	(0.53,1.25)		1.03	(0.66, 1.62)	
Linear		0.93	(0.70, 1.23)	0.6	0.91	(0.66,1.25)	0.6	0.86	(0.64, 1.17)	0.4
**PTB**		**1^st^ month**			**1^st^ trimester**			**1 week before birth** a		
		**OR**	**(95% CI)**	**p**	**OR**	**(95% CI)**	**p**	**OR**	**(95% CI)**	**p**
**adjusted:**		**month, year**			**month, year**			**month, year, gestational** **age, day of week**		
Quintile										
	1	1		0.6	1		0.06	1		0.4
	2	0.94	(0.86, 1.02)		1.00	(0.92, 1.09)		1.04	(0.96, 1.12)	
	3	0.95	(0.84, 1.06)		0.91	(0.79, 1.04)		0.97	(0.87, 1.07)	
	4	0.93	(0.79, 1.09)		1.01	(0.85, 1.20)		0.97	(0.84, 1.12)	
	5	0.91	(0.75, 1.10)		0.92	(0.75, 1.13)		0.93	(0.79, 1.08)	
Linear		0.94	(0.85, 1.04)	0.2	0.97	(0.84, 1.12)	0.7	1.00	(0.93, 1.08)	1

a pregnancies-at-risk approach.

## Results

Baseline data for both states is shown in Table 1.

### Results of Analyses

In both states more babies were born between July and September and more babies conceived between October and December.

A weak seasonal pattern was also observed for tLBW in Brandenburg (Table 2). The risk was 19% higher (95% CI: 5%, 35%, p = 0.02, note: p-values (Wald-test) are calculated for a difference between all months/all seasons) for babies conceived in spring compared to summer and 15% higher (CI: 2%, 30%, p = 0.1) for those born in winter compared to summer. The examination of a seasonality of PTB in Brandenburg showed some slight increased risk for conceptions in spring. The same analyses in Saxony did not lead to significant findings. Even though there was a strong non-linear (U-shaped) association between maternal age and PTB and tLBW with the highest risk in the youngest age group, we found only very weak confounding of the association between season and PTB or tLBW by maternal age. Furthermore, there was no change in the pattern of the odds ratios and they changed less than one percent when adjusting for maternal age.

**Table 4:** Association between temperature and tLBW or PTB in Saxony.

tLBW		1^st^ trimester			2^nd^ trimester			3^rd^ trimester		
		OR	(95% CI)	p	OR^1^	(95% CI)	p	OR	(95% CI)	p
adjusted:		month, year			month, year			month, year		
			
Quintile										
	1	1		0.06	1		0.4	1		0.2
	2	0.99	(0.86, 1.14)		1.09	(0.94,1.28)		1.09	(0.93, 1.28)	
	3	0.91	(0.73, 1.14)		0.99	(0.78,1.26)		1.10	(0.84, 1.43)	
	4	0.97	(0.72, 1.31)		0.92	(0.67,1.26)		0.86	(0.60, 1.23)	
	5	0.74	(0.51, 1.06)		1.02	(0.70,1.50)		0.92	(0.61, 1.40)	
Linear		0.89	(0.70, 1.12)	0.3	1.09	(0.82,1.45)	0.5	1.15	(0.87, 1.52)	0.3
**PTB**		**1^st^ month**			**1^st^ trimester**			**1 week before birth** a		
		**OR**	**(95% CI)**	**p**	**OR**	**(95% CI)**	**p**	**OR**	**(95% CI)**	**p**
**adjusted:**		**month, year**			**month, year**			**month, year, gestational** **age, day of week**		
Quintile										
	1	1		0.4	1		0.09	1		0.8
	2	1.01	(0.94,1.09)		0.93	(0.87, 1.01)		1.01	(0.94, 1.09)	
	3	0.98	(0.87, 1.09)		0.99	(0.88, 1.11)		1.06	(0.96, 1.18)	
	4	1.07	(0.92, 1.25)		1.02	(0.87, 1.19)		1.04	(0.92, 1.18)	
	5	1.11	(0.92, 1.32)		1.13	(0.92, 1.37)		1.04	(0.90, 1.19)	
Linear		1.03	(0.94, 1.14)	0.5	1.06	(0.94, 1.21)	0.3	1.00	(0.94, 1.07)	1

a pregnancies-at-risk approach.

The analysis of an association between temperature and tLBW or PTB showed at most weak associations that differed considerably between states (Tables 3 and 4) or in main compared to sensitivity analysis were season was modeled as B-splines with different degrees of flexibility (not shown in tables).

The model deviance residuals were plotted over time and showed no obvious pattern. No temporal autocorrelation of the exposure effect or over-dispersion of the data was apparent.

**Figure 1:**
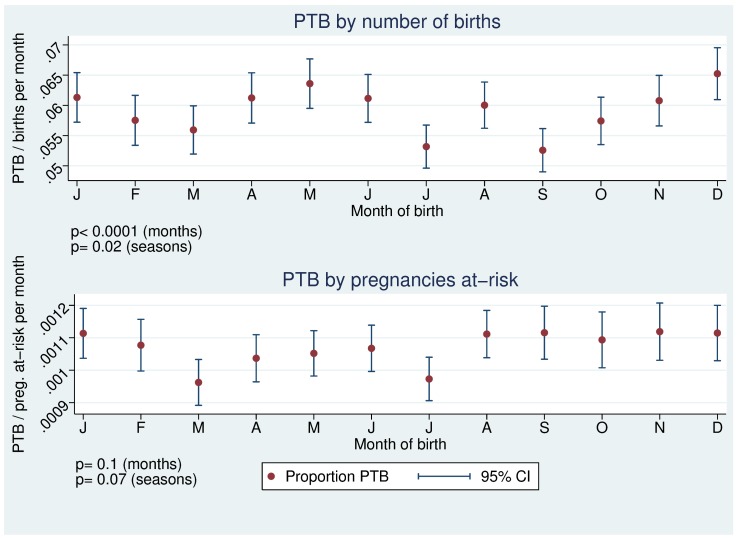
Seasonal pattern of PTB without and with pregnancies-at-risk approach.

**Figure 2:**
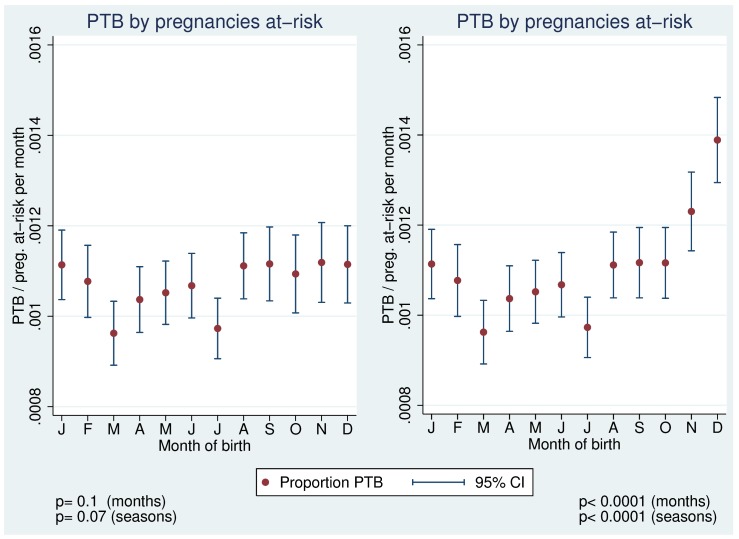
PTB proportions with (left) and without (right) data restriction (pregnancies-at-risk approach).

### Methodological Observations

The described analyses involved substantial potential for biased results in terms of artificial associations with temperature or spurious seasonal patterns. In the following paragraphs we will compare those results, describe their developments and how to avoid them. Distortions of effects were consistently stronger in Saxony, mainly due to a shorter observation period. Because of this and for ease of understanding, comparisons are shown for Saxony only. However, those biases were also apparent for Brandenburg.

For analyses by date of conception, data needed to be restricted at the beginning of the observation period to avoid preponderance of longer and at the end to avoid preponderance of shorter pregnancies [46]. Without restriction very strong (p<0.0001 and p = 0.001) evidence for an association between temperature in the 1st month and 1st trimester and the risk of PTB would have appeared with a higher risk for conceptions in the two warmest temperature quintiles (OR 1.24, CI: 1.05, 1.48 and OR 1.25, CI: 1.03, 1.53). Without avoiding selection by the length of pregnancies at both ends of data collection, shorter or longer pregnancies can be falsely associated with exceptional hot or cold temperature periods if such periods happen to occur at the start or end of data collection. This selection of pregnancies at the ends of data collection can also lead to an artificial seasonal pattern when longer pregnancies at the start or shorter pregnancies at the end of the study are associated with a particular season. However, the introduction of an artificial seasonality will usually be avoided when the first day of the study is one calendar date after the day on which the study ends (regardless of year, like in this study with start 1st January and end 31st December). In this case, the preponderance of short and long pregnancies will tend to cancel each other out. This is not the case for temperature which can vary a lot between the same season in different years. (45).

To examine PTB by date of birth only pregnancies at-risk to be born preterm on that day were considered. If, as in analyses for tLBW, number of births had been taken instead, evidence for a stronger seasonality with peaks of PTB in May and December would have been observed for Saxony after adjusting for year, gestational age in weeks and day of the week (difference between seasons p = 0.02, difference between months p<0.0001, Figure 1). No spurious association between temperature in the last week before birth (the only temperature period that was analysed by date of birth) and PTB appeared when number of births was used as denominator instead of number of pregnancies at-risk.

At the end of the dataset increasingly fewer pregnancies were at-risk to end preterm as pregnancies that were born after the end of the study period did not appear in the risk-set. Very low numbers in the denominator to calculate PTB-proportions are a potential source of bias as few PTBs at the end of data collection gain much greater importance. For a constant denominator at the end of the dataset, the last 15 weeks of data were excluded. Artificially high proportions of PTB in November and December and very strong evidence for a seasonal pattern of PTB (p<0.0001) would have resulted otherwise (Figure 2). No association between temperature in the week before birth and PTB was introduced when the dataset was not restricted at the end.

## Discussion

Weak evidence for a seasonal pattern with an increased risk for conceptions in spring and births in winter was observed for both conditions. However, the level of evidence differed considerably between the two states. An even weaker and inconsistent association between temperature and tLBW or PTB was detected.

However, many prior studies described significant seasonal variations of (t)LBW or PTB with remarkable differences between regions [6]. Peaks of LBW [50], [51] or PTB [14], [17], [21], [52], [53] were frequently observed in autumn and winter. Conceptions in February were more likely to be preterm [54]. As discussed many studies also found different associations of adverse pregnancy outcome and temperature [7], [19]–[23], [25], [26]. These studies showed a lot of heterogeneity in the methods they applied. Some assumed a linear effect of temperature and used different methods for seasonal adjustment [19]–[23], [25], [26]. Other examinations detected no association between temperature and adverse pregnancy outcome [55]–[58]. As most of these studies which analysed time-series data did not explicitly adjust for any of the three possible biases that are described in this study, it is likely that at least some of the heterogeneity arises from this.

Some limitations of our study can be identified. The data was routinely collected and may therefore contain errors [55], [59]. Mistakes in gestational age estimation can introduce random misclassification of outcome and an underestimation of the association. Temperature exposure was assumed to be equal across one state. To justify this, data from eight stations showed high similarity and correlation. Still, urban temperatures can be considerably higher than those in the countryside [60]. Furthermore, temperature exposure varies inter-individually and especially pregnant women may spend a lot of time indoors [55]. On the other hand, young women usually show high mobility and the place where they give birth might differ from residence during pregnancy [61].

A constrained lag model was used to investigate temperature. We considered temperature averaged over different time periods of pregnancy (trimester, first month, or last week as specified in the method section), to allow for both acute and variously delayed effects.

Season was examined as smooth splines, Fourier or indicator terms. Indicator terms have advantages in interpretability but apply simplifying assumptions like a consistent effect across and the same length for each month [62]. Smooth splines convey a more flexible approach and take a time-trend into account. However, the chosen flexibility of the curve is a trade-off between optimal control and following the data too close [63].

No adjustment was made for air pollution, hypertensive disorders of pregnancy or infections. We did not have data on these factors, but if we did it is not clear whether they should be adjusted for. Air pollution and preeclampsia have been shown to be associated with both adverse pregnancy outcome and temperature [18], [64]–[66], but as air pollution and preeclampsia might both be on the causal pathway between temperature and adverse pregnancy outcome [6], [67], [68] arguably no adjustment should be made. A similar argument applies to adjusting seasonal patterns for infections, as infections show seasonal patterns [69], [70] and can initiate PTB. They might therefore be on the causal pathway for a seasonal pattern of PTB [71], [72].

The strengths of this analysis include a large sample size, a long time-span and the possibility for comparisons between two states. An important advantage of the time-series design is the inherent control for non-time-varying individual risk factors, even unknown or unrecorded [55], [73].

As already highlighted, in analyses of adverse pregnancy outcome artificial seasonal patterns might occur e.g. a truncation bias [46] and the necessity to apply a pregnancies-at-risk approach [15]. Another potential for biased results arose when the number of pregnancies at-risk was not balanced at the end of the dataset. An otherwise ever decreasing denominator would have resulted in an overestimation of PTB-rates at the end of the year and would have introduced a strong seasonality (Figure 2). Failure to account for these potential sources of biased results can lead to spurious associations with temperature or seasons and might potentially explain some of the heterogeneous findings of the past. This is especially true for PTB and for LBW when no differentiation is made between term and LBW-babies that are born preterm.

This study might have implications for similar studies on environmental exposures on pregnancy outcome as sources for spurious seasonal patterns are discussed and appropriate approaches demonstrated.
